# Relationship between Anemia and Anthropometric Profile in Tabari Cohort Population: A Case-Control Study

**DOI:** 10.34172/jrhs.2025.183

**Published:** 2025-04-01

**Authors:** Ali Moghadami, Akbar Hedayatizadeh-Omran, Motahareh Kheradmand, Mahmood Moosazadeh

**Affiliations:** ^1^Student Research Committee, Faculty of Medicine, Mazandaran University of Medical Sciences, Sari, Iran; ^2^Gastrointestinal Cancer Research Center, Non-communicable Diseases Institute, Mazandaran University of Medical Sciences, Sari, Iran; ^3^Health Sciences Research Center, Mazandaran University of Medical Sciences, Sari, Iran

**Keywords:** Anemia, Anthropometric indices, Tabari cohort study

## Abstract

**Background:** Anemia is a common blood disorder in developing countries and is associated with diseases such as diabetes, as well as cardiovascular and pulmonary diseases. This study aimed to investigate the relationship between anemia and anthropometric profiles in the Tabari cohort population.

**Study Design:** A case-control study.

**Methods:** In this study, we used a subset of data collected during the first phase of the Tabari cohort study (TCS). All participants who had anemia were included in the case group (1352 individuals) using the census method. The control group (1352 individuals) consisted of participants who did not have anemia and were randomly selected from the remaining participants. The case and control groups were matched for age and gender. Anthropometric indices, including body mass index (BMI), waist circumference (WC), and waist-to-hip ratio (WHR), were measured by qualified and trained persons. Hematological indices were measured, and data were analyzed using the chi-square test and independent *t* test. A multiple logistic regression analysis was used to adjust for possible confounding variables.

**Results:** The place of residence, education level, socioeconomic status, WC, BMI, and WHR were significantly different between the case and control groups (*P*<0.05). Anthropometric indices, including BMI (adjusted OR=0.75; 95 % CI 0.61, 0.91), WC (adjusted OR=0.86; 95 % CI 0.74, 1.00), and WHR (adjusted OR=0.75; 95 % CI 0.64, 0.88), were significantly different between the case and control groups (*P*<0.05).

**Conclusion:** Anthropometric indices were associated with anemia, and participants with higher BMI, WC, and WHR were less likely to develop anemia.

## Background

 Anemia is a global health problem. The World Health Organization (WHO) defines anemia as a hemoglobin level of < 12 g/dL in non-pregnant women and < 13 g/dL in men, or an insufficient number and reduced size of red blood cells.^1,2^ It affects more than 27% of the world’s population. This disorder is more prevalent in low-income and middle-income countries. The incidence of anemia in Iran is estimated to be approximately 8.83 %. This rate is higher among children, women, and older adults.^3,4^ Anemia increases the risk of mortality and other related co-morbidities.^5^ Iron and folate deficiency, renal failure, and unexplained reasons are listed as causes of anemia.^6^ The early diagnosis of anemia improves the treatment process and prevents complications. One indicator of anemia is anthropometric indices. These parameters are simple, non-invasive, and cost-effective and can be measured easily.^7,8^

 Associations between anthropometric indices and hypertension,^8^ metabolic syndrome,^9^ and diabetes mellitus^10^ have been reported in previous studies. Findings showed that high waist circumference (WC) and waist-to-height ratio (WHtR) are associated with diabetes and hypo-HDL cholesterolemia. Greater body mass index (BMI) and WHtR are associated with hypertension^8-10^; however, the relationship between anthropometric indices and anemia in adults has not been extensively investigated. Therefore, we aimed to investigate the relationship between anemia and anthropometric indices in a case-control study.

## Materials and Methods

###  Study design and participants 

 In the present case-control study, we utilized a subset of data collected in the first phase of Tabari cohort study (TCS). TCS was a population-based cohort study conducted in Sari, Mazandaran, Iran. TCS is part of a nationwide cohort called Prospective Epidemiological Research Studies in Iran (PERSIAN), which was carried out in different parts of Iran. In the first phase of the PERSIAN cohort study, 180 000 Iranians, aged 35-70 years, from 18 geographically distinct areas in Iran were recruited. TCS was conducted in Mazandaran province of Iran. Mazandaran province is a district in northern Iran, located in the foothills of the Alborz mountain range, which includes mountains and plains. The capital city of Mazandaran province (Sari) and a mountainous region (Kiasar) were selected to conduct TCS. Between June 2015 and November 2017, 10255 men and women (35-70 years of age) were enrolled from the aforementioned regions. The rationale, objectives, and design of the PERSIAN cohort and TCS have been published.^11-13^ In this study, all participants who were anemic by definition were included using the census method as a case group (1352 individuals). The control group consisted of participants without anemia who were randomly selected from the remaining participants (1352 individuals). The case and control groups were matched for age and gender. The exclusion criterion was a history of malignancy or myocardial infarction. Anemia was defined as hemoglobin < 13.0 g/dL and < 12.0 g/dL in men and women, respectively.^1^

###  Anthropometric indices 

 Qualified and trained individuals were responsible for measuring the anthropometric indices. They followed a standardized methodology. Weight was measured using a calibrated balance scale (SECA 755; SECA, Hamburg, Germany). A stadiometer (SECA 226, Hamburg, Germany) was used to measure height, and the participants were asked to stand with their arms by their sides, take off their shoes, and put their feet together. Waist and hip circumferences were measured according to the National Health and Nutrition Examination Survey protocol.^14^ BMI, WC, and waist-to-hip ratio (WHR) were used as the anthropometric indices.

###  Data collection 

 A structured questionnaire standardized by the PERSIAN cohort team was used to collect information. The questionnaire consisted of items on demographic information, socioeconomic status, occupational history, type of fuel used, lifestyle, reproductive history, history of chronic diseases, family history, oral health, sleep status, physical activity, smoking and drinking habits, food frequency, food supplements, drinking habits, dietary habits, and exposure to pesticides. Blood samples were collected from all participants in the TCS after 12 hours of fasting, and hematological indices were measured using an automated cell counter (Nihon Kohden, Tokyo, Japan).

###  Statistical analysis

 Statistical analyses were performed using SPSS version 25.0. Qualitative and quantitative variables between the two groups with and without anemia were compared using chi-square and independent *t*-tests, respectively. Multiple logistic regression analysis was used to determine the relationship between anemia and anthropometric profile. Among all the demographic and clinical variables (residence, marital status, socioeconomic level, educational level, MET, diabetes, renal failure, rheumatoid arthritis, hypothyroidism, and heart failure), those with a significance level less than 0.25 were entered into the model in order to adjust their effect on the relationship between anemia and anthropometric profile. All tests were two-tailed, and *P* values of less than 0.05 were considered statistically significant.

## Results

 Among all participants of the TCS, 1352 participants were anemic and assigned to the case group. Table 1 presents a comparison of demographic and clinical variables between the two groups. Except for marital status (*P* = 1.000), all demographic variables, including place of residence (*P* < 0.001), educational level (*P* < 0.001), socioeconomic status (*P* < 0.001), and MET (*P* < 0.001), were significantly different between the case and control groups. In contrast, a history of diabetes, kidney failure, hypothyroidism, and heart failure did not differ between the two groups. The history of rheumatoid arthritis was significantly different between the two groups (*P* = 0.048).

**Table 1:** Comparison of demographic and clinical variables between case and control groups

**Variables**	**Case group**	**Control group**	***P *** **value**
Residence			
Urban	1180	904	0.001
Mountainous areas	172	448	
Marital status			
Married	1231	1232	1.000
Single or divorced	121	120	
Socioeconomic level			
5 (highest)	344	344	0.001
4	341	341	
3	288	288	
2	234	234	
1 (lowest)	145	312	
Educational level (year)			
College	383	299	0.001
9-12	424	371	
6-8	155	141	
1-5	257	354	
No education	133	187	
MET			
< Median	823	670	0.001
≥ Median	529	682	
Diabetes			
Yes	237	227	0.646
No	1115	1125	
Renal failure			
Yes	26	19	0.367
No	1326	1333	
Rheumatoid arthritis			
Yes	70	48	0.048
No	1282	1304	
Hypothyroidism			
Yes	185	160	0.166
No	1167	1192	
Heart failure			
Yes	132	110	0.157
No	1220	1242	

 The comparison of anthropometric indices between the case and control groups is presented in Table 2. BMI (*P* = 0.007), WC (*P* = 0.059), and WHR (*P* < 0.001) were significantly different between the case and control groups. Findings showed that the number of participants with BMI ≥ 30 in the control group was greater than that in the case group (18.7% vs. 17.5%). Moreover, the number of men and women with WC > 102 cm and ≥ 88 cm, respectively, was higher in the control group (28% vs. 26.1%).

**Table 2:** Comparison of anthropometric indices in the case and control groups

**Variables**	**Case, n=1352**	**Control, n=1352**	***P *** **value**
Body mass index (kg/m^2^)			
< 25	346	277	0.007
25-29.9	533	570	
≥ 30	473	505	
Waist circumference (cm)			
Men < 102, women < 88	645	595	0.059
Men ≥ 102, women ≥ 88	707	757	
Waist-hip ratio			
Men < 0.9, women < 0.85	491	404	0.001
Men ≥ 0.9, women ≥ 0.85	861	948	

 Univariate and multiple logistic regression revealed significant relationships between anemia and BMI, WC, and WHR (Table 3). In the univariate and multiple logistic models, BMI < 25 was considered as a reference. According to multiple logistic regression, participants with a BMI of 25-29.9 were 36% less likely to be anemic than those with a BMI of < 25 (*P* < 0.05), and participants with a BMI of ≥ 30 were 40% less likely to be anemic. The risk of being anemic in participants with a WC ≥ 102 or 88 cm was 19% lower than that in the reference group (*P* = 0.012). Moreover, participants with WHR ≥ 0.9 or 0.85 were 25% less likely to be anemic than the reference group (*P* = 0.001).

**Table 3:** Relationship between anthropometric profile and anemia using univariate and multiple logistic regression analyses

**Variables**	**Unadjusted OR (95% CI)**	***P *** **value**	**Adjusted OR (95% CI)**^a^	***P *** **value**
Body mass index (kg/m^2^)				
< 25	Ref.		Ref.	
25-29.9	0.75 (0.61, 0.91)	0.004	0.64 (0.52, 0.78)	0.001
≥ 30	0.75 (0.61, 0.92)	0.005	0.60 (0.48, 0.74)	0.001
Waist circumference (cm)				
Men < 102, women < 88	Ref.		Ref.	
Men ≥ 102, women ≥ 88	0.86 (0.74, 1.00)	0.054	0.81 (0.69, 0.94)	0.012
Waist-hip ratio				
Men < 0.9, women < 0.85	Ref.		Ref.	
Men ≥ 0.9, women ≥ 0.85	0.75 (0.64, 0.88)	0.001	0.75 (0.63, 0.89)	0.001

^a^ Adjusted for area of residence, socioeconomic level, education level, physical activity, hypothyroidism, rheumatoid arthritis, and heart failure.

## Discussion

 The findings of this study showed that anthropometric indices were significantly different between participants with and without anemia. After adjusting for possible confounding variables, significance remained consistent. Interestingly, our findings showed a protective role for high BMI, WC, and WHR in anemia. In addition, the results of our study showed that residents of mountainous regions were significantly more likely to be anemic than urban residents.

 Consistent with our findings, previous studies have shown that people with higher BMI, WC, and WHR are less likely to develop anemia,^15^ and the lowest rate of anemia was observed in obese people.^16^ Results of a study conducted by Jamshidi et al in Iran showed a positive relationship between abdominal obesity but not general obesity and total iron-binding capacity, HB, HCT, and ferritin (*P* < 0.001).^17^ In contrast, Ghaemi et al showed that iron levels were below normal in 56.1% of children with obesity.^18^ The difference between our results and those of Hoang et al^15^ and Ghaemi et al^19^ could be attributed to the age of the study population, as evidence suggests that long-term obesity in the early stages of life is associated with iron deficiency. Therefore, screening for anemia in children with high BMI can be effective in reducing the prevalence of anemia. Inconsistent with our findings, some studies reported that iron deficiency was significantly more common in obese and overweight people.^3,20-23^ A history of iron supplementation and the control of the underlying disease can better explain this relationship.

 A comparison of urban and rural data showed that the prevalence of anemia in rural people was significantly higher. Inequalities in education, available facilities, and fertility preferences increase the risk of anemia in rural populations. In the current study, educational level and socioeconomic status were significantly different between cases and controls. In contrast to our study, no demographic or socioeconomic factors were associated with any type of anemia in the study by Hoang et al.^15^

 Despite the large sample size of the present study, there are still some limitations. We did not assess the type of anemia in the study population, which can prevent this study from being compared with similar studies. The results of this study cannot be generalized to age groups of < 35 years. Another limitation of the present study is the nature of case-control studies that prevents the causal relationship between anthropometric indices and anemia.

**Highlights**

- Anemia was more prevalent in residents of mountainous areas.
- There was a significant association between anthropometric indices and anemia.
- Participants with higher body mass index (BMI), waist circumference (WC), and waist-to-hip ratio (WHR) were less likely to have anemia.

## Conclusion

 According to the results, anthropometric measurements were associated with anemia; therefore, individuals with higher BMI, WC, and WHR were less likely to have anemia.

## Acknowledgments

 This study was supported by the Iranian Ministry of Health and the Research Deputy of Mazandaran University of Medical Science. We would like to thank all the participants and staff of the PERSIAN and Tabari cohort team.

## Competing Interests

 The authors declare no conflict of interests.

## Ethical Approval

 This study was approved by the Ethics Committee of Mazandaran University of Medical Sciences (IR.MAZUMS.IMAMHOSPITAL.REC.1400.069).

## Funding

 Research Deputy of Mazandaran University of Medical Science supported the study.
